# Dose-guided individualized planning target volume margin optimization in nasopharyngeal carcinoma: a retrospective megavoltage computed tomography–based cumulative analysis

**DOI:** 10.3389/fonc.2025.1727150

**Published:** 2026-01-16

**Authors:** Xingxing Yuan, Longfei Xu, Changfei Gong, Junming Jian, Wenheng Zheng, Changwei Luo, Yun Zhang

**Affiliations:** 1 Department of Radiation Oncology, Jiangxi Cancer Hospital & Institute (The Second Affiliated Hospital of Nanchang Medical College), Nanchang, Jiangxi, China; 2 Jiangxi Clinical Research Centre for Cancer, Nanchang, Jiangxi, China; 3 Key Laboratory of Personalized Diagnosis and Treatment of Nasopharyngeal Carcinoma, Nanchang, Jiangxi, China

**Keywords:** cumulative dose, dose-guided adaptive radiotherapy, megavoltage computed tomography, nasopharyngeal carcinoma, planning target volume margin, tomotherapy

## Abstract

**Background:**

In radiotherapy for nasopharyngeal carcinoma (NPC), balancing target coverage with protection of adjacent organs remains challenging. This study aimed to evaluated the cumulative dose deviations resulting from different planning target volume (PTV) margins during tomotherapy (TOMO) using megavoltage computed tomography (MVCT) and investigated the feasibility of individualized margin optimization, with the goal of minimizing volume and protecting surrounding tissues.

**Methods:**

Forty patients with NPC treated with TOMO were retrospectively analyzed. Daily MVCT scans were acquired throughout treatment. Using deformable image registration, the dose from each fraction was mapped to the planning CT, and cumulative doses were reconstructed using MIM software. For each patient, isotropic 1–4 mm virtual expansions were applied to the gross tumor volume (GTV) and clinical target volume (CTV) to generate “virtual” structures. These were evaluated for V100% dose coverage under the accumulated dose. Planned and accumulated doses were compared across expansion margins using V100%, D95%, conformity index, and homogeneity index, with differences assessed by paired t-tests. Dose variations in organs at risk (OARs) were also assessed.

**Results:**

Planned and accumulated V100% doses were within 5% for most targets, however, certain virtual expansions demonstrated detectable dose discrepancies. For GTVnx, a +2 mm margin significantly reduced accumulated V100% (96.55% ± 3.04%) compared with the planned dose (99.18% ± 0.73%; p < 0.001). For GTVnd, both +1 mm and +2 mm margins significantly reduced accumulated V100% (99.21% ± 1.17% and 99.20% ± 1.17%, respectively; p < 0.01). For CTV1, a +2 mm margin yielded higher accumulated V100% (99.57% ± 1.49%) than planned (99.89% ± 0.21%; p = 0.212), suggesting deformation-related over-coverage. Larger margins (3–4 mm) reduced coverage, though not significantly. For CTV2, accumulated V100% remained consistent across all margins. The +1 mm expansion produced a small but significant increase (99.72% ± 0.38% vs 99.99% ± 0.23%; p = 0.006), while larger expansions (2–4 mm) had no effect, suggesting 1–2 mm may be optimal.

**Conclusion:**

MVCT-based cumulative dose evaluation provides a more accurate assessment of the delivered dose than conventional geometric methods. Individualized, dose-driven PTV margin strategies may improve tumor coverage while minimizing OAR exposure, thereby advancing precision radiotherapy for NPC.

## 1 Introduction

Nasopharyngeal carcinoma (NPC) is a highly aggressive head and neck malignancy for which radiotherapy remains the primary curative treatment (1–8). Owing to its complex anatomical location and its proximity to critical structures such as the brainstem, spinal cord, and optic apparatus, precise dose delivery is essential to achieve tumor control while minimizing radiation-induced toxicity (9–11). Tomotherapy (TOMO), which integrates intensity-modulated radiotherapy with daily megavoltage computed tomography (MVCT) image guidance, provides excellent dose conformity and homogeneity, making it particularly well-suited for NPC treatment (12–14). Daily MVCT improves setup accuracy and enables dose reconstruction with consistent calculation accuracy, whereas cone-beam CT (CBCT) is limited by imaging artifacts and less consistent HU-dose conversion (15, 16). Studies have shown that MVCT-based dose calculations can achieve dose deviations within ±1% compared to the planning CT, whereas CBCT-based calculations may deviate by 3–5% in heterogeneous regions (17).

According to ICRU principles, the expansion from the gross tumor volume (GTV) to the clinical target volume (CTV) accounts for possible subclinical extension along anatomical routes, modulated by natural tissue barriers. In contrast, the expansion from CTV to the planning target volume (PTV) is intended to compensate for uncertainties associated with treatment setup and internal motion, thereby ensuring adequate CTV coverage throughout treatment delivery. In current clinical practice, uniform isotropic planning target volume (PTV) margins of 3–8 mm are routinely used to account for setup errors and anatomical variations (18, 19). However, this “one-size-fits-all” approach presents a clinical dilemma: while larger margins mitigate the risk of target underdosing, they also increase unnecessary irradiation of adjacent organs at risk (OARs). For example, reducing PTV margins in NPC has been reported to lower the mean dose to the parotid glands by 7.1–7.7%, spinal cord D1% by 2.6%, and brainstem D1% by 2.3%, without compromising target coverage (20). Despite these potential benefits, existing PTV margin designs are still predominantly based on population-level geometric uncertainties or rigid image registration (21–23), both of which present two major limitations. First, these approaches lack dosimetric validation; whether a prescribed margin ensures adequate cumulative dose coverage throughout treatment remains largely unverified (24, 25). Anatomical changes during the treatment course can progressively reduce target coverage (e.g., PTV2 coverage decreased by 3.01%) and increase the OAR doses (e.g., parotid mean dose increased by 2.45 Gy), effects that daily repositioning alone cannot fully correct (26). Second, they do not adequately account for target-specific dynamics such as tumor regression or variable motion patterns. For instance, lower cervical lymph nodes may require margins of up to 3 mm, whereas primary gross tumor volumes (GTVs) may require only 1 mm (27, 28).

Deformable image registration (DIR) combined with daily MVCT enables cumulative dose reconstruction that captures the actual delivered dose distribution across the entire treatment course (23, 29–31). This dose-guided radiotherapy approach provides a direct dosimetric basis for individualized PTV margin optimization, overcoming the limitation of purely geometric surrogates. By linking margin size to verified cumulative dose coverage, optimal margins can be identified that ensure tumor control while minimizing the risks of OAR toxicity, such as xerostomia and hearing loss (32, 33).

The optimal PTV margins varied according to target type when evaluated against the accumulated dose, and individualized margin reduction proved feasible without compromising coverage. We therefore conducted a quantitative evaluation of cumulative dose deviations resulting from different PTV margins in patients with NPC undergoing TOMO with MVCT guidance. In this study, we used MVCT-based DIR to reconstruct cumulative doses for NPC patients treated with TOMO, simulated isotropic expansions ranging from +1 mm to +4 mm for GTVs and clinical target volumes (CTVs), and evaluated whether V100% thresholds (≥ 99% for GTV, ≥ 98% for CTV) were achieved in each scenario. To the best of our knowledge, this is the first study to integrate MVCT-based cumulative dose reconstruction with systematic isotropic margin simulation in NPC, thereby providing a clinically applicable framework for dose-driven, patient-specific PTV optimization.

## 2 Methods

### 2.1 Patient selection

This retrospective study included 40 patients with histologically confirmed NPC who underwent definitive TOMO as the primary treatment at Jiangxi Cancer Hospital between 2021 and 2024. The study protocol was reviewed and approved by the Institutional Review Board of Jiangxi Cancer Hospital (Approval No. 2022ky216). Informed consent was waived due to the retrospective nature of the study, and all patient data were de-identified prior to analysis. The clinicopathological characteristics are detailed in **Table 1**.

**Table 1:** Detailed clinicopathological characteristics.

Category	Number (n)	Percentage (%)
Gender		
Male	30	75
Female	10	25
Age (years)		
Median (range)	55 (24–77)	
T stage		
T1	5	12.5
T2	10	25
T3	15	37.5
T4	10	25
N stage		
N0	5	12.5
N1	10	25
N2	15	37.5
N3	10	25
M stage		
M0	35	87.5
M1	5	12.5
Clinical stage		
I	2	5
II	5	12.5
III	18	45
IV	15	37.5

### 2.2 Immobilization and treatment planning

All the patients were immobilized in the supine position using a thermoplastic head-neck-shoulder mask with a foam cushion (Klarity, China) to ensure reproducible positioning. Simulation CT scans (Siemens SOMATOM Definition AS) were performed from 2 cm superior to the frontal sinus to 3 cm inferior to the clavicle, acquiring contiguous 3-mm slices, an in-plane resolution of 512 × 512, and a voxel size of 1.0 × 1.0 × 3.0 mm³. Daily image guidance was provided using MVCT (TOMO system) with a slice thickness of 2 mm. These parameters ensured consistent geometric accuracy and reproducibility of DIR-based dose accumulation.

In accordance with Report 83 of the International Commission on Radiation Units and Measurements and the clinical guidelines for NPC management issued by the Chinese Society of Clinical Oncology, two radiation oncologists independently delineated target volumes. These included the primary nasopharyngeal tumor (GTVnx) and metastatic cervical lymph nodes (GTVnd). The first CTV (CTV1) was generated by expanding the GTVnx by 8 mm to encompass the high-risk areas surrounding the nasopharynx. The second CTV (CTV2) was defined by expanding CTV1 by 5 mm to include anatomical regions such as the nasal cavity, maxillary sinus mucosa, and retropharyngeal lymph nodes (34, 35). PTVs were then generated by isotropically expanding the GTVs by 3 mm and the CTVs by 5 mm. For margin-reduction analysis, additional isotropic expansions were generated: +1 mm and +2 mm for GTVs and +1 mm, +2 mm, +3 mm, and +4 mm for CTVs. These expansions were chosen to systematically evaluate a range from aggressive reduction to moderate expansion relative to our clinical standards (3 mm for GTV, 5 mm for CTV) and were denoted as “virtual structures.” To avoid confusion, we defined “PGTV” as the conventional GTV + 3 mm volume and “virtual GTV + X mm” or “virtual CTV + X mm” as the simulated expansions.

Plans were generated using the Accuray Precision Treatment Planning System (version 1.1.1; Accuray, Sunnyvale, CA, USA) with a helical delivery technique. The key planning parameters included a 6-MV flattening filter-free(6-FFF) photon beam energy, a 2.5-cm field width, 2.5–3.0 dynamic jaw mode modulation factor, and a pitch of 0.287–0.43. Prescribed doses to PGTVnx, PGTVnd, PTV1, and PTV2 were 70 Gy, 66–70 Gy, 60 Gy, and 54–55 Gy, respectively, delivered in 30–33 fractions. Dose normalization required that ≥95% of each PTV volume received 100% of the prescribed dose (V100% ≥ 95%). Institutional standards further required ≥99% coverage for GTVnx/GTVnd and ≥98% for CTV1/CTV2, with ≤5% of PTVnx and PTVnd volumes exceeding 107% of the prescribed dose. Doses to the OARs were minimized as previously described (36).

Cumulative dose reconstruction was performed using MIM Maestro software (version 7.1.3; MIM Software Inc., Cleveland, OH, USA). DIR was conducted with a voxel-based free-form deformation algorithm based on mutual information. Deformation accuracy was validated by landmark-based evaluation (<2 mm average error) and by Jacobian determinant analysis to ensure physiologic, non-folding deformation fields. The maximum landmark deviation was 3.5 mm, observed near the parapharyngeal soft-tissue interface, and no negative Jacobian determinants were found in the deformation vector fields used for dose mapping. The accumulated dose was calculated by deformably mapping the daily MVCTs onto the planning CT.

Dose-volume histograms (DVHs) were generated for all PTVs and OARs. Primary dosimetric endpoints included V100%, D95%, and D98% for targets, and mean or maximum doses for OARs, in accordance with Quantitative Analyses of Normal Tissue Effects in the Clinic and institutional constraints. The homogeneity index (HI = [D2% – D98%]/D50%) (35) and conformity index (CI = [VRI/TV] × [VRI/VRI(total)]) (37) were also calculated but were reported only as secondary reference parameters, as the primary focus of this study was cumulative dose coverage and OAR sparing.

### 2.3 Accumulated dose calculation

**Figure 1:**
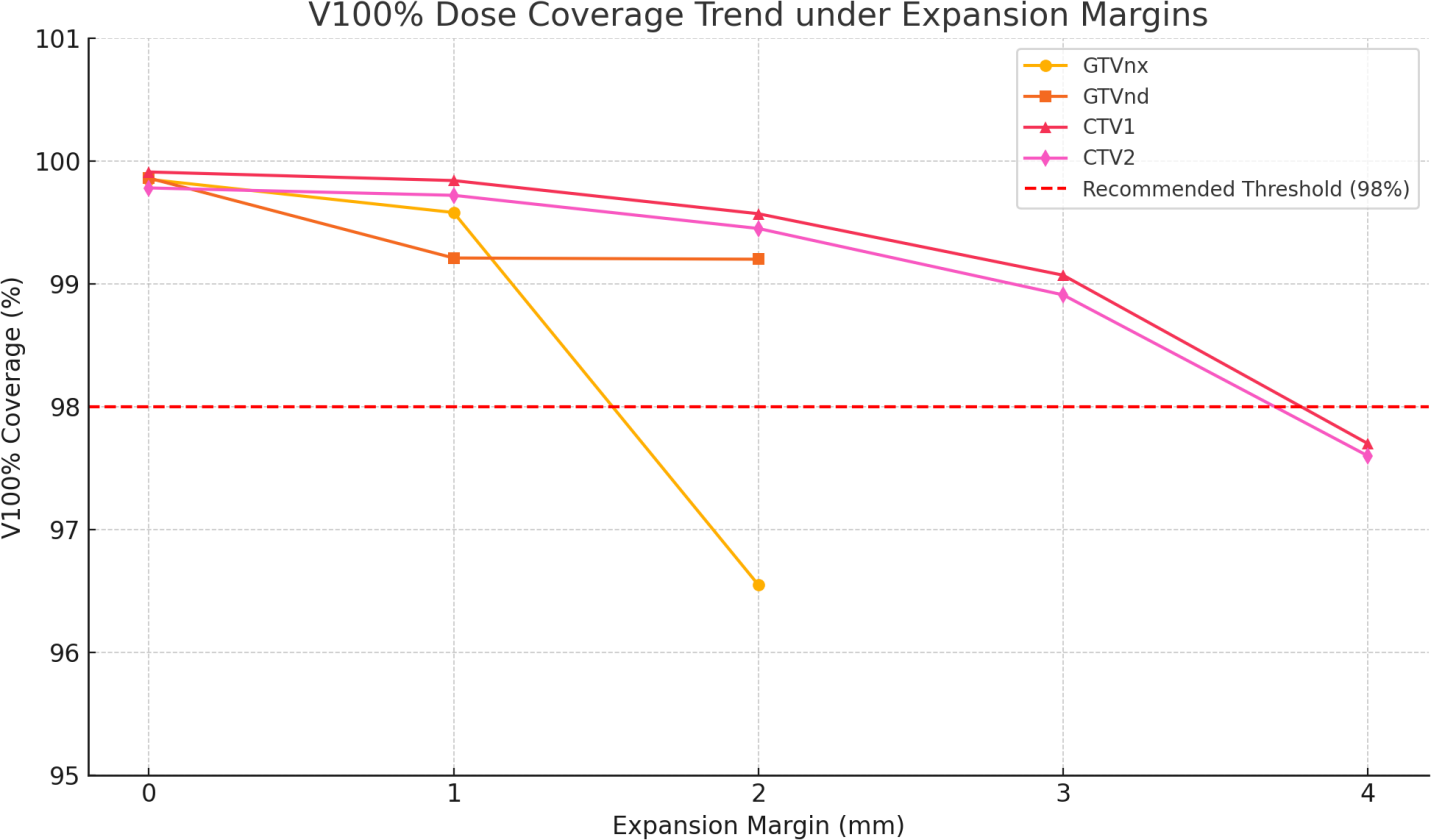
Workflow of MVCT-guided dose accumulation in TOMO.

Before each treatment fraction, daily MVCT scans with coverage identical to that of the planning CT were acquired and registered using a bone- and soft-tissue matching algorithm, focusing on the nasopharynx and cervical spine. Translational and roll deviations were corrected with couch shifts before irradiation, whereas larger discrepancies triggered manual repositioning and rescanning. Delivered dose data were extracted from treatment sinograms, and both MVCT images and dose files were imported into MIM Maestro software (version 7.1.3) for DIR and cumulative dose reconstruction. Fractional doses were deformably mapped onto the planning CT geometry on a voxel-by-voxel basis, and all fractions were subsequently summed to obtain an individualized cumulative dose distribution. This enabled retrospective dosimetric evaluation of target and OAR doses under real-world anatomical and positional variations (**Figure 1**).

Step 1: Daily MVCT acquisition and registration

Prior to each treatment fraction, MVCT scans were obtained within the same anatomical range as the planning CT scans to ensure consistency. MVCT images were automatically registered to the planning CT scan using a bone- and soft-tissue algorithm focused on the nasopharyngeal and cervical spine regions. Translational and roll deviations were corrected, whereas pitch and yaw rotations were not compensated. Pitch and yaw were not mechanically compensable. If residual deviations exceeded institutional limits (≤ ± 3 mm translation, ≤ ± 2° rotation), patients were repositioned and rescanned. Across all patients, the mean ± SD setup errors were 1.4 ± 1.3 mm (X), –0.8 ± 2.1 mm (Y), and 1.4 ± 1.5 mm (Z); rotational errors were 0.0 ± 0.1° (pitch), 0.1 ± 1.5° (yaw), and 0.0 ± 0.1° (roll). The average correction vector was 1.4 ± 0.8 mm, and 4.2% of fractions required manual repositioning. The immobilization system demonstrated high reproducibility, with post-correction residual errors consistently within these tolerance limits and no significant impact on overall workflow efficiency.Final bone-soft-tissue matching was visually verified by a senior therapist prior to beam-on.

Step 2: Patient positioning correction and verification

Couch shifts based on the registration results were applied before irradiation to minimize setup errors. The initial MVCT-to-CT registrations were reviewed and approved by experienced radiation oncologists and senior therapists. Daily alignments were performed by trained therapists, with periodic audits by senior staff to ensure inter-operator consistency.

Step 3: Dose extraction and data import

The delivered dose information for each fraction was extracted from the treatment sinogram files. Both MVCT images and their corresponding dose files were imported into MIM Maestro (version 7.1.3; MIM Software Inc., Cleveland, OH, USA), ensuring that anatomical variations and machine delivery records were integrated for subsequent analysis. For every MVCT scan, the dose was recomputed from the corresponding machine-logged sinogram. After post-processing corrections, the reconstructed dose grids were assigned to their MVCT images, which were then deformably registered to the original planning CT. no MVCT fractions were excluded. Accumulation was completed by voxel-wise deformation of these dose matrices via DIR, following MIM’s validated workflow.

Step 4: DIR-based dose mapping

DIR was applied to map each fraction’s voxel-by-voxel dose distribution onto the planning CT geometry. This approach captured treatment-related anatomical changes such as tumor regression, parotid shrinkage, and body weight loss, thereby ensuring spatially accurate comparison with the planned dose. Quality assurance of DIR was performed through visual inspection of the deformation vector fields and landmark-based checks on selected slices. The deformation vector fields and DIR accuracy metrics were independently reviewed by two senior physicists to guarantee reproducibility. No non-physical (negative) Jacobian regions were detected, and validation tests confirmed that differences in MVCT slice thickness did not introduce observable deformation artifacts.All acquired MVCT fractions were included in the analysis; none were excluded due to image quality or registration failure, ensuring the cumulative dose reflected the complete delivered treatment.

Step 5: Cumulative dose reconstruction and evaluation

All DIR-mapped fractional doses were summed across the entire treatment course to generate individualized accumulated dose distributions. These cumulative distributions allowed for the retrospective evaluation of the delivered doses to both target volumes and OARs under real-world anatomical and positional variations. DVHs derived from the accumulated doses were compared with the planned values to identify clinically significant deviations.

Paired-sample t-tests were used to evaluate the differences between the planned and accumulated doses for all target volumes and OARs. For marginal expansion simulations, repeated-measures one-way ANOVA was performed to assess the changes in V100% coverage across different isotropic expansion levels (+0 mm to +4 mm). All statistical analyses were conducted using SPSS software (version 24.0; IBM Corp., Armonk, NY, USA). Statistical significance was defined as p < 0.05. All statistical analyses were reviewed by an experienced biostatistician to ensure methodological accuracy and reproducibility.

## 3 Results

Substantial anatomical changes were observed throughout the treatment course. The mean volume reduction for GTVnx and GTVnd was 18.5% ± 10.2% and 22.3% ± 12.1%, respectively. The mean body weight loss was 3.2% ± 2.5%. Parotid gland volume decreased by an average of 24.7% ± 8.9% (left) and 25.4% ± 9.3% (right). These changes were tracked using the daily MVCT and incorporated into the DIR process.

**Figure 2:**
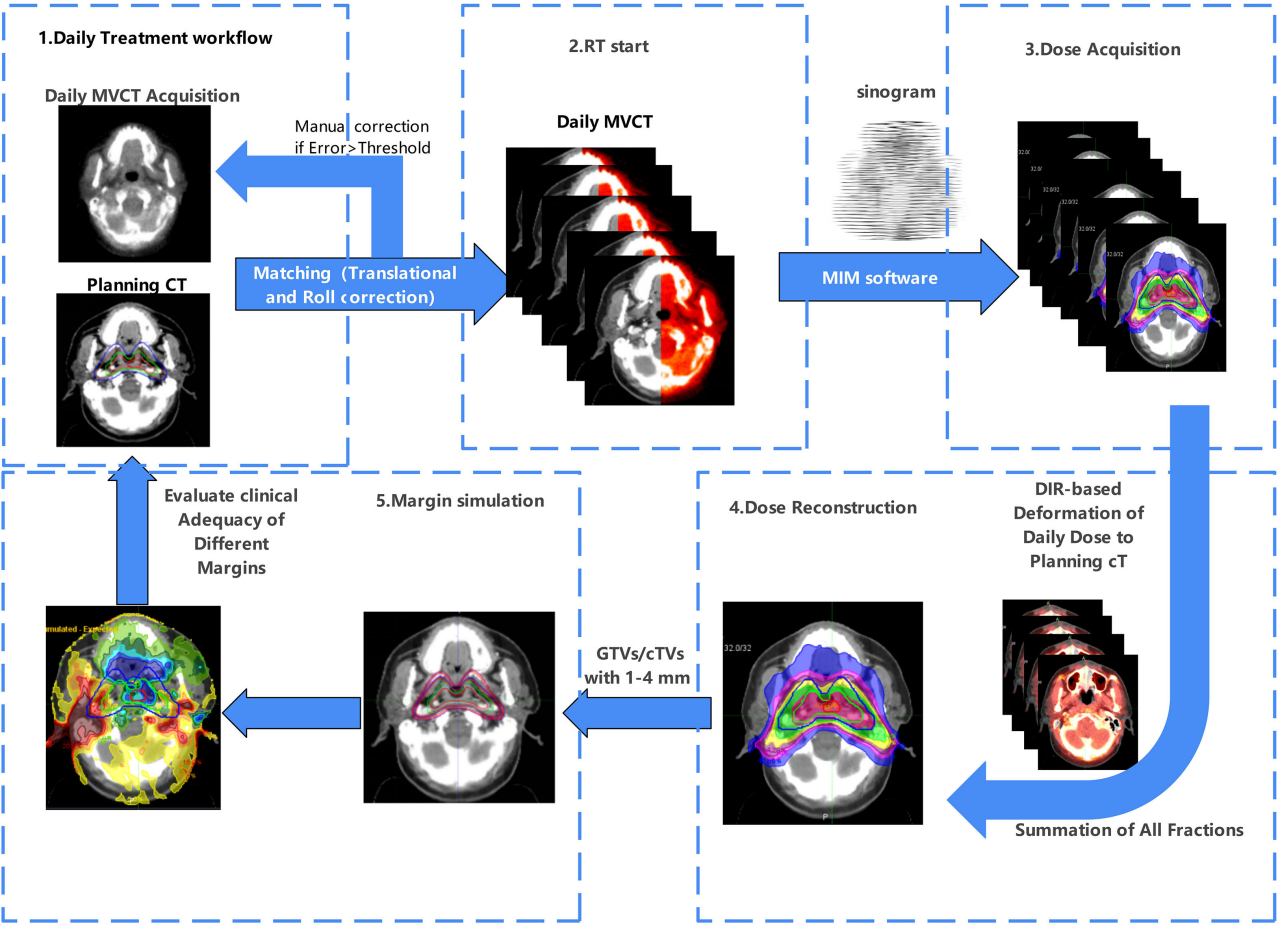
A direct comparison of dose distribution between planned and accumulated plans for one patient: **(a)** Dose difference map (Accumulated–Planned), highlighting regions of underdosage (blue) and overdosage (red), particularly around the lateral margins of the PTVs and in the ipsilateral parotid gland. **(b)** Planned dose distribution based on the original treatment plan, showing ideal target coverage and OAR sparing. **(c)** Accumulated dose distribution reconstructed using deformable image **(d)** Dose–volume histograms (DVHs) comparing planned (solid lines) and accumulated (dashed lines) doses for GTVs, CTVs, PTVs, and selected OARs. Deviations between planned and delivered doses highlight the clinical importance of cumulative dose analysis for evaluating margin adequacy and organ-at-risk protection.

**Figure 2** illustrates a representative patient (male, 58 years, T3N2M0) selected for having median values of tumor volume change (-15.2%) and body weight loss (-3.8%) during treatment, thus reflecting the cohort’s central tendency. Significant dose deviations were observed primarily at the target volume periphery, with the maximum point dose difference at the PTVnx periphery being -4.5 Gy. The V100% for PGTVnx decreased from 97.1% (planned) to 92.0% (accumulated).These discrepancies reflected the anatomical changes during treatment, and were most prominent in the posterior-lateral aspect of the target, correlating with parotid gland medial shift and neck contour reduction observed on serial MVCTs. Notable differences were also seen in adjacent OARs, particularly in the parotid glands and oral cavity. From the DVHs, clear differences between the accumulated and planned doses were noted in the PTVnx, PTV1, GTVnx, and CTV1. In normal tissues, dose discrepancies were particularly prominent in the right parotid gland and lens.

The dosimetric differences between the planned and accumulated doses for the target volumes and OARs are shown in **Tables 2** and **3**. Overall, the discrepancies between the planned and accumulated doses were minimal for the GTVs and CTVs, indicating a relatively stable dose delivery in core tumor regions. However, more pronounced reductions were observed in the PTVs, particularly PGTVnx and PTV1. For PGTVnx, the accumulated V100%, D95%, and D98% decreased by −4.16%, −0.79 Gy, and −1.11 Gy, respectively, all statistically significant (p < 0.001). A similar trend was noted for PTV1, with reductions of −1.19%, −0.40 Gy, and −0.62 Gy (p < 0.01). These findings suggest that the peripheral margins of PTVs may be vulnerable to underdosage, potentially owing to daily setup errors, anatomical changes, or deformable registration inaccuracies. In contrast, the dosimetric parameters for GTVnx, GTVnd, CTV1, and CTV2 remained relatively stable, with differences between the planned and accumulated doses falling within clinically acceptable ranges. For instance, the reductions in V100% for GTVnx and CTV1 were less than 0.5%, and the mean dose difference was within 0.2 Gy. For most OARs, the differences between planned and accumulated doses were not statistically significant. However, several structures—including the lenses, parotid glands, right middle ear, right inner ear, and oral cavity—showed significant increases in the accumulated dose. The maximum doses to the left and right lenses increased by 0.63 Gy and 0.67 Gy, respectively (p < 0.01). The mean doses to the left and right parotid glands rose by 1.26 Gy and 2.38 Gy, respectively (p < 0.001). Additionally, the right middle ear and inner ear exhibited increases of 0.54 Gy and 0.79 Gy in mean dose (p < 0.001), while the oral cavity showed an increase of 0.52 Gy (p < 0.001). These changes may be attributed to cumulative positioning errors, anatomical deformation during treatment, or inadequacies in margin design, underscoring the need for dose-guided adaptation to enhance OAR protection.

**Table 2:** Dosimetric parameter results comparisons between planned and accumulated plans for targets.

Target volume		Parameters	Plan dose	Accumulated dose	Difference (Acc - Plan)	P-value
PGTVnx		V100% (%)	96.68 ± 1.34	92.52 ± 4.37	-4.16	**0.000**
		D95% (Gy)	70.37 ± 0.34	69.58 ± 0.88	-0.79	**0.000**
		D98% (Gy)	69.57 ± 0.52	68.46 ± 1.06	-1.11	**0.000**
		Dmean (Gy)	72.12 ± 0.41	71.90 ± 0.56	-0.22	**0.002**
PGTVnd		V100% (%)	97.69 ± 1.67	97.13 ± 2.20	-0.56	0.210
		D95% (Gy)	69.25 ± 1.49	69.06 ± 1.57	-0.19	0.506
		D98% (Gy)	68.65 ± 1.45	68.24 ± 1.58	-0.41	0.139
		Dmean (Gy)	70.96 ± 1.59	70.91 ± 1.67	-0.05	0.861
PTV1		V100% (%)	98.49 ± 1.32	97.30 ± 1.78	-1.19	**0.000**
		D95% (Gy)	61.33 ± 0.82	60.93 ± 0.96	-0.40	**0.001**
		D98% (Gy)	60.44 ± 0.79	59.82 ± 0.97	-0.62	**0.000**
		Dmean (Gy)	67.88 ± 1.29	67.69 ± 1.32	-0.19	**0.013**
PTV2		V100% (%)	97.88 ± 1.13	97.16 ± 1.57	-0.72	**0.000**
		D95% (Gy)	55.03 ± 0.48	54.84 ± 0.65	-0.19	**0.001**
		D98% (Gy)	53.94 ± 0.79	53.41 ± 1.11	-0.53	**0.000**
		Dmean (Gy)	62.07 ± 1.23	62.04 ± 1.37	-0.03	0.519
		CI	0.80 ± 0.144	0.78 ± 0.14	-0.02	**0.000**
GTVnx		V100% (%)	99.85 ± 0.38	99.61 ± 0.86	-0.24	0.028
		D95% (Gy)	71.39 ± 0.46	71.25 ± 0.58	-0.14	0.078
		D98% (Gy)	71.09 ± 0.49	70.92 ± 0.62	-0.17	0.082
		Dmean (Gy)	72.42 ± 0.50	72.32 ± 0.56	-0.10	0.086
GTVnd		V100% (%)	99.86 ± 0.60	99.83 ± 0.59	-0.03	0.845
		D95% (Gy)	70.28 ± 1.63	70.21 ± 1.73	-0.07	0.808
		D98% (Gy)	70.05 ± 1.64	69.96 ± 1.72	-0.09	0.761
		Dmean (Gy)	71.33 ± 1.64	71.18 ± 1.80	-0.15	0.641
CTV1		V100% (%)	99.91 ± 0.19	99.87 ± 0.34	-0.04	0.361
		D95% (Gy)	64.57 ± 24.34	64.18 ± 22.70	-0.39	**0.002**
		D98% (Gy)	63.58 ± 21.70	63.12 ± 20.03	-0.46	**0.002**
		Dmean (Gy)	69.88 ± 15.09	69.70 ± 15.35	-0.18	**0.012**
CTV2		V100% (%)	99.78 ± 0.31	99.72 ± 0.38	-0.06	0.336
		D95% (Gy)	56.03 ± 13.32	56.14 ± 13.25	0.11	0.284
		D98% (Gy)	55.52 ± 12.38	55.60 ± 12.12	0.08	0.292
		Dmean (Gy)	64.12 ± 16.42	64.15 ± 17.88	0.03	0.313
GTVnx	+1 mm	V100% (%)	99.93 ± 0.15	99.58 ± 0.86	-0.35	**0.007**
	+2 mm	V100% (%)	99.18 ± 0.73	96.55 ± 3.04	-2.63	**0.000**
GTVnd	+1 mm	V100% (%)	99.86 ± 0.69	99.21 ± 1.17	-0.65	**0.000**
	+2 mm	V100% (%)	99.74 ± 0.29	99.20 ± 1.17	-0.54	**0.010**
CTV1	+1 mm	V100% (%)	99.90 ± 0.14	99.84 ± 0.50	-0.06	0.346
	+2 mm	V100% (%)	99.89 ± 0.21	99.57 ± 1.49	-0.32	0.212
	+3 mm	V100% (%)	99.38 ± 0.66	99.07 ± 1.52	-0.31	0.215
	+4 mm	V100% (%)	97.76 ± 1.64	97.70 ± 1.92	-0.06	0.814
CTV2	+1 mm	V100% (%)	99.99 ± 0.23	99.72 ± 0.38	-0.27	**0.006**
	+2 mm	V100% (%)	99.73 ± 0.56	99.45 ± 0.46	-0.33	0.155
	+3 mm	V100% (%)	99.14 ± 0.69	98.91 ± 1.16	-0.23	0.183
	+4 mm	V100% (%)	97.53 ± 1.39	97.60 ± 1.41	0.07	0.837

Dosimetric values are presented as mean ± standard deviation. “Planned Dose” refers to values calculated from the initial treatment planning system, while “Accumulated Dose” is derived from deformable image registration of daily MVCT scans. Differences were calculated as (Accumulated Dose − Planned Dose). Statistical significance was evaluated using paired t-tests; bolded p-values indicate p < 0.05. According to institutional protocol, V100% ≥ 98% for GTV and ≥ 98% for CTV were used as coverage thresholds. Failures to meet these criteria are considered potential underdosage. These findings provide evidence for evaluating the robustness of current margin design and support individualized margin adjustment based on cumulative dose performance.

**Table 3:** Dosimetric parameter results comparisons between planned and accumulated plans for OARs.

OAR	Parameters	Planned Dose (Gy)	Accumulated Dose (Gy)	Difference (Gy)	Difference (%)	P-value
Brain stem	Max	52.72 ± 6.56	52.77 ± 6.98	0.05	0.09%	0.869
Brain stem	Mean	25.12 ± 4.45	25.02 ± 4.37	-0.1	-0.40%	0.265
Spinal Cord	Max	30.35 ± 5.18	30.40 ± 5.37	0.05	0.16%	0.732
Optic Chiasm	Max	36.79 ± 18.21	37.64 ± 18.74	0.85	2.26%	0.556
Optic Nerve L	Max	34.39 ± 18.27	34.95 ± 18.43	0.56	1.60%	0.239
Optic Nerve R	Max	36.72 ± 18.89	37.08 ± 19.04	0.36	0.97%	0.476
Lens L	Max	4.99 ± 1.78	5.62 ± 2.08	0.63	11.21%	**0.000**
Lens R	Max	5.13 ± 1.81	5.80 ± 2.41	0.67	11.55%	**0.001**
Parotid L	Mean	31.19 ± 3.87	32.45 ± 4.80	1.26	3.88%	**0.000**
Parotid R	Mean	31.90 ± 4.13	34.28 ± 4.81	2.38	6.94%	**0.000**
Submandibular L	Mean	52.40 ± 6.80	52.62 ± 6.98	0.22	0.42%	0.255
Submandibular R	Mean	52.72 ± 6.73	53.01 ± 6.60	0.29	0.55%	0.098
Middle Ear L	Mean	43.10 ± 7.32	43.44 ± 7.42	0.34	0.78%	0.019
Middle Ear R	Mean	43.56 ± 9.56	44.10 ± 9.62	0.54	1.22%	**0.000**
Inner Ear L	Mean	63.13 ± 6.31	63.54 ± 6.39	0.41	0.65%	0.021
Inner Ear R	Mean	63.13 ± 6.35	63.92 ± 6.37	0.79	1.24%	**0.000**
Larynx​	Mean	34.16 ± 9.37	34.28 ± 9.53	-0.12	0.35%	0.041
Thyroid​	Mean	46.04 ± 4.30	46.13 ± 4.37	-0.09	0.20%	**0.003**
Oral Cavity​	Mean	35.64 ± 8.25	35.12 ± 9.26	0.52	0.99%	**0.000**
T Lobe L​	Mean	11.85 ± 6.00	11.78 ± 5.95	0.07	0.59%	0.101
T Lobe R​	Mean	11.72 ± 5.77	11.65 ± 5.73	0.07	0.60%	0.055

Values are expressed as mean ± standard deviation. Planned doses represent values from the original radiotherapy plan; accumulated doses are derived from deformable summation of daily MVCT-guided adaptive dose recalculations. The “Difference” refers to the accumulated dose minus the planned dose. Paired t-tests were used to assess statistical significance, with p < 0.05 considered statistically significant (bolded). Dose increases in organs at risk (OARs) may indicate unintended irradiation due to anatomical variations or setup uncertainties. These results underscore the importance of adaptive planning and cumulative dose monitoring in clinical practice.

Subgroup analysis revealed that patients with weight loss >5% (n=8) experienced larger reductions in PTV1 V100% (mean -2.3% vs. -1.0% in weight-stable patients, p=0.021). Similarly, cases with initial GTVnx volume >40 cm³ (n=12) showed greater PGTVnx D98% decrease (-1.8 Gy vs -0.9 Gy, p=0.045). We defined a clinically significant deviation as a reduction in V100% of ≥3% for PTVs or an increase in mean dose of ≥2 Gy for serial OARs (e.g., spinal cord). By this criterion, 7 patients (17.5%) had clinically significant PTV undercoverage.

### 3.1 Dose coverage analysis of the GTV and CTV under simulated margin expansion

To further assess the appropriateness of the current margin expansion strategies, isotropic expansions of 1–4 mm were retrospectively applied to GTVs and CTVs. The resulting accumulated V100% coverage was analyzed to evaluate the dose conformity across varying margin levels.

### 3.2 GTV coverage analysis

GTVnx: Under a +2 mm expansion, the accumulated V100% decreased to 96.55% (p < 0.001), falling below the institutional threshold of 98%. This suggests that excessive margin expansion may compromise the dose conformity at the tumor periphery, likely due to the complex anatomy or interfractional deformation.

GTVnd: The accumulated V100% remained above 99.2% under expansions of +1 mm and +2 mm. Although statistically significant (p < 0.05), these differences were likely clinically negligible, indicating robust geometric consistency in this region.

### 3.3 CTV coverage analysis

CTV1: The accumulated V100% remained above 98% across all margin conditions. A slight overcoverage was noted at +2 mm (99.57%, p < 0.05), whereas a gradual decline was observed at +3 mm and +4 mm, suggesting that excessive margins may result in anatomical mismatch or inefficient dose distribution.

CTV2: The most stable dose coverage was observed for CTV2. The accumulated V100% peaked at 99.72% under the +1 mm expansion (p < 0.01) and remained consistently above 98% across all margin levels. Further expansion did not yield additional benefits and may increase unnecessary exposure of the surrounding normal tissues.

**Figure 3:**
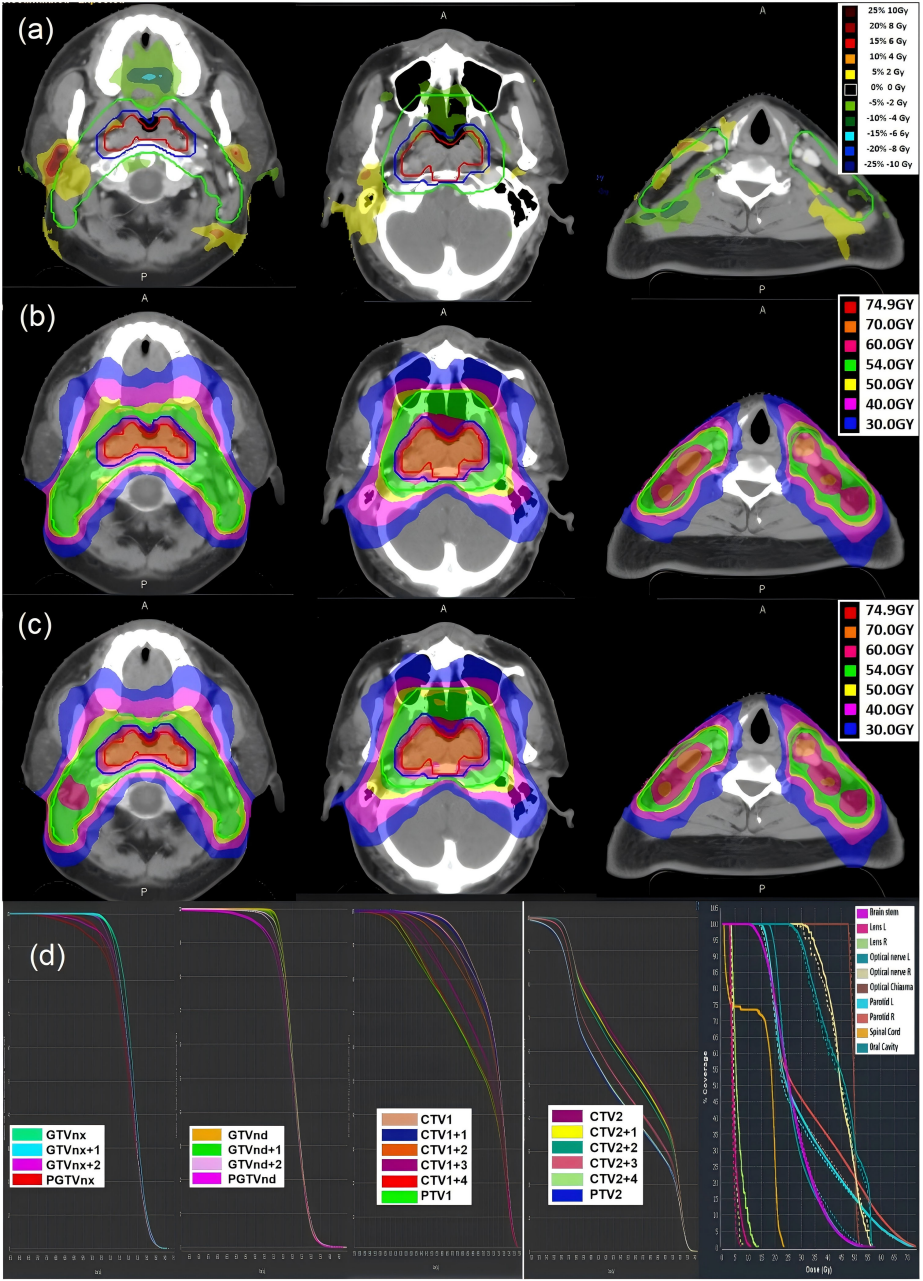
Trends in V100% dose coverage across varying expansion margins for GTVs and CTVs. Abbreviations are used per medical writing conventions: GTV (Gross Tumor Volume); CTV (Clinical Target Volume); V100% (Volume receiving 100% of the prescription dose).

As shown in **Figure 3**, the V100% dose coverage for GTVs and CTVs demonstrates a consistent trend under different margin expansions, suggesting that a uniform margin expansion approach may not be optimal for all target volumes. Specifically, a +2 mm expansion significantly compromises peripheral dose conformity and should be applied with caution. For CTV2, the observed geometric stability indicates that margin reduction may be feasible to minimize the dose to adjacent normal structures. Both GTVnd and CTV1 show stable dose coverage; however, individualized evaluation based on patient-specific anatomy and motion characteristics remains necessary.

## 4 Discussion

This study systematically evaluated the differences between planned and accumulated doses in patients with NPC treated with TOMO, using daily MVCT imaging and DIR via the MIM system. By focusing on GTV and CTV expansions, we quantitatively analyzed whether various isotropic margin settings (≥1–2 mm) were sufficient to achieve the required V100% dose coverage, thereby assessing the feasibility of individualized margin design in clinical practice. We found that most target volumes and OARs demonstrated dose deviations within 5%, reflecting high dosimetric consistency under TOMO guidance with daily MVCT.

However, GTVnx and CTV1 exhibited significant declines in cumulative V100% when expanded by 2 mm, suggesting that excessive margin expansion may paradoxically result in peripheral dose degradation, jeopardizing full target coverage. In contrast, GTVnd and CTV2 maintained robust dose coverage even with larger expansions, This inherent “dosimetric robustness” can be attributed to their favorable anatomical context: GTVnd, located in the neck, is constrained by more homogeneous soft tissues and coupled to bony setup landmarks, while CTV2, as a larger elective volume, naturally smooths out local perturbations through its size.These findings suggest that the margins can be reduced without compromising tumor control, thereby minimizing unnecessary OAR exposure.Therefore, a universal expansion approach appears suboptimal; instead, Our results support a risk-adapted expansion strategy, especially for complex skull-base tumors with a stable anatomy.

Based on our findings, we propose a preliminary framework for individualized margins: 1) For GTVnx in patients with minimal baseline motion (e.g., early T-stage, no parapharyngeal extension), consider reducing the PTV margin from 3 mm to 1–2 mm. 2) For CTV2, which showed high dosimetric robustness, a reduction from 5 mm to 3–4 mm appears feasible. 3) Adaptive replanning or margin re-evaluation should be triggered if weekly dose accumulation reveals a V100% drop of >3% for any PTV, or if anatomical changes (e.g., weight loss >5%, parotid volume reduction >20%) are detected on MVCT.From a translational perspective, this study emphasizes that dose adequacy—not merely geometric setup correction—should serve as the central criterion for PTV margin design, particularly in high-precision systems such as TOMO. Reliance solely on population-based geometric margins may no longer be sufficient in the era of image-guided adaptive radiotherapy. MVCT-based dose accumulation enables the retrospective validation of treatment fidelity and promotes the clinical adoption of personalized margin optimization.

Our study builds upon and extends the growing body of literature exploring adaptive planning and margin evaluation in head and neck radiotherapy. While prior studies have investigated setup errors, anatomical variations, and adaptive radiotherapy (ART) strategies, few have systematically examined the impact of different isotropic PTV margins on cumulative dose delivery using daily MVCT-guided deformable dose accumulation, especially in NPC. Yao et al. (26) retrospectively analyzed 16 NPC patients treated with helical TOMO and investigated the relationship between rotational and residual setup errors and dose deviation using MIM software. Their results indicated that rotational errors, particularly pitch and roll, had a significant dosimetric impact on the average doses to PTVnd, CTV1, and CTV2, whereas translational errors showed no clear correlation with dose deviation. Although informative, their study primarily addressed the mechanical origins of dose variance rather than margin adequacy. Simopoulou et al. (27) conducted a comprehensive review of ART in head and neck cancer, confirming its positive impact on both dosimetric precision and toxicity reduction. However, most of the available evidence was derived from CBCT-based verification or plan recalculation models rather than actual dose accumulation. Our use of daily MVCT-based deformable dose reconstruction offers a more accurate, real-world depiction of dose delivery over time and highlights the potential for integrating margin evaluation within ART protocols. Rudat et al. (30) explored the influence of the online verification frequency on the patient setup errors and safety margins. They found that even with daily image-guided radiotherapy, over 10% of patients with head and neck cancer exhibited residual errors exceeding 5 mm, underscoring the limitations of rigid setup correction alone. However, an important technical limitation must be acknowledged. Although daily MVCT guidance effectively reduces translational setup uncertainties, rotational deviations—particularly pitch and yaw—were not corrected in the tomotherapy platform used in this study. This is an inherent mechanical constraint of the system and is shared by other ring-gantry–based units such as Varian Ethos, which also lack six-degree-of-freedom correction capability. Uncompensated rotations may lead to misalignment of elongated structures along the cranio-caudal axis and contribute to peripheral dose degradation in complex regions such as the skull base. While the magnitude of rotational errors in our cohort was generally within previously reported ranges, their potential influence on cumulative dose delivery should be recognized. Future margin-evaluation studies incorporating systems with full 6DoF correction may help clarify the relative contribution of rotational errors to PTV undercoverage.

In addition, although this study leveraged MVCT-based dose accumulation—which benefits from improved geometric stability and reduced HU variability compared with CBCT—it is important to consider the broader applicability of our findings. The core concept of dose-guided margin evaluation is not limited to MVCT platforms and may be adapted by institutions using CBCT-based IGRT. However, CBCT images present challenges such as greater HU instability, increased susceptibility to artifacts, and larger uncertainties in dose recalculation and DIR performance. These factors may influence the accuracy of cumulative dose reconstruction and thus require appropriate correction strategies, such as CBCT-to-CT HU mapping, scatter correction, or physics-informed DIR algorithms. Therefore, while the margin-reduction principles identified in our study may serve as a conceptual framework, CBCT-guided workflows would require dedicated validation to ensure equivalent dose accuracy before implementing similar individualized PTV reductions in clinical practice.

Our study complements these prior findings by employing daily MVCT to reduce positioning uncertainty and by utilizing DIR-based dose accumulation to expose hidden discrepancies in target and OAR dosing, even under apparently correct alignments. Hazeral et al. (33) recently analyzed 30 head and neck cancer patients undergoing ART due to anatomical changes and weight loss. They emphasized that frequent ART replanning improves cumulative PTV coverage and OAR sparing, particularly when weight loss exceeds 4% per week. While their analysis centered on the timing and frequency of ART interventions, our work complements this by identifying specific anatomical regions and margin scenarios most susceptible to cumulative dose degradation, thereby offering a practical roadmap for preemptive plan adaptation. Although previous studies have addressed the setup errors, ART benefits, and imaging frequency, none have quantitatively compared cumulative dose outcomes across multiple PTV margin scenarios in NPC. Our study is the first to integrate MVCT-based dose accumulation with isotropic margin modeling, thereby providing novel insights into individualized, dose-guided radiotherapy planning in clinical practice.

This research presents several methodological and clinical strengths. Daily MVCT acquisition enables accurate tracking of anatomical changes throughout the treatment course. Deformable dose accumulation using MIM software permitted realistic reconstruction of the delivered doses, surpassing the limitations of rigid dose summation. Analysis of multiple isotropic expansions (1–4 mm) across both GTVs and CTVs allowed for a nuanced evaluation of margin sufficiency and redundancy. A parallel analysis of the accumulated doses to OARs highlights the need for improved sparing strategies, particularly for auditory and salivary structures. To the best of our knowledge, this is the first study to associate MVCT-derived cumulative dose distributions with individualized PTV margin evaluations in NPC, representing a novel contribution to precision radiotherapy research. Despite these strengths, some limitations must be acknowledged. First, this was a retrospective study, and no adaptive replanning was performed during the treatment process; thus, clinical interventions could not be implemented to correct underdosage. Second, although 40 patients were included, a larger cohort would increase statistical power and enhance generalizability. Third, margin expansions were uniformly applied (e.g., isotropic +1 mm or +2 mm), whereas in clinical practice, direction-specific margins (e.g., asymmetric margins) may yield better results. Fourth, the anisotropic slice thickness between the planning CT (3 mm) and the daily MVCT (2 mm) may present a potential source of uncertainty for the deformable image registration (DIR) process, particularly for curved anatomical surfaces. While rigorous visual inspection and landmark verification were employed to ensure registration quality, the specific impact of this mismatch on deformation accuracy and dose-mapping precision was not quantitatively isolated in this study and warrants further investigation. Fifth, the study used consensus target contours; interobserver variability in GTV/CTV delineation, which contributes to the overall margin uncertainty, was not quantified. Future studies incorporating multiple observers and probabilistic margin approaches would be valuable to account for this factor.Sixth.although we documented dosimetric changes, their direct impact on local control or toxicity outcomes was not evaluated.

Importantly, the dosimetric trends observed in this study should be viewed as hypothesis-generating. Their clinical significance requires prospective validation through studies comparing long-term outcomes—such as local control and treatment-related toxicities—between patients treated with optimized versus conventional PTV margins.

To build upon these findings, future studies should prospectively incorporate ART workflows that modify PTV margins in response to real-time imaging and dose trends. Additional directions include non-isotropic margin modeling tailored to local anatomical changes and organ motion, integration of MRI or PET/CT fusion for more precise GTV/CTV delineation, AI-driven margin optimization algorithms using accumulated dose deviation data, and multicenter validation to enhance generalizability and inform margin selection guidelines for NPC.

## 5 Conclusions

This study combined MVCT-based cumulative dose evaluation with isotropic margin modeling in patients with NPC treated using TOMO. Although GTVs and CTVs demonstrated stable dose delivery, PTVs—particularly the PGTVnx—were more vulnerable to underdosing. For example, the GTVnx V100% decreased to 96.55% under a +2 mm expansion, falling below the 98% adequacy threshold. OAR structures, such as the parotid glands, lenses, ears, and oral cavity, received higher cumulative doses with larger expansions. These findings emphasize the importance of margin adaptation. MVCT-based dose accumulation supports an individualized, dose-guided approach to PTV margin determination. This strategy can help secure adequate tumor coverage while minimizing unnecessary OAR exposure, thereby advancing precision radiotherapy for patients with NPC.

## Data Availability

The raw data supporting the conclusions of this article will be made available by the authors, without undue reservation.
